# Optimizing nitrogen reduction for improved grain yield and nitrogen-use-efficiency in two rice genotypes grown in hilly regions of Sichuan

**DOI:** 10.3389/fpls.2026.1833459

**Published:** 2026-05-13

**Authors:** Peng Ma, Xiaohong Qin, Jianli Chai, Ran Wang, Lubing Jia, Xiaotian Jiang, Guotao Yang, Yungao Hu

**Affiliations:** College of Life Science and Agri-forestry, Southwest University of Science and Technology, Mianyang, China

**Keywords:** enzyme activity, grain yield, nitrogen reduction, nitrogen-use-efficiency, rice, sugar content

## Abstract

**Introduction:**

The hilly region of Sichuan is a core rice-producing area in Southwest China.Long-term excessive fertilization has resulted in low nitrogen-use-efficiency and aggravated agricultural non-point source pollution, which seriously restricts the green and high-yield development of rice production.

**Methods:**

To determine the optimal nitrogen reduction rate for nitrogen-efficient rice varieties in this region and to reveal the regulatory effects of nitrogen reduction on rice yield, nitrogen utilization, and physiological metabolism, a two-year field experiment was conducted using two rice cultivars, Byou 3611 and Yixiangyou 2115. Four nitrogen treatments were applied: 180 kg·hm^-2^ (N3), 150 kg·hm^-2^ (N2), 120 kg·hm^-2^ (N1), and 0 kg·hm^-2^ (N0, control). Yield, dry matter accumulation and translocation, nitrogen-use-efficiency,sugar content, and key enzyme activities involved in sucrose metabolism were systematically measured.

**Results:**

The results showed that 150 kg·hm^-2^ nitrogen (N2) was the optimal treatment, with the highest grain yield in both years. The yields of Byou 3611 and Yixiangyou 2115 reached 12101.05 kg·hm^-2^ and 11941.19 kg·hm^-2^, respectively, which were significantly higher than those under conventional nitrogen application (N3). Excessive nitrogen failed to increase yield but reduced the seed-setting rate, whereas large reduction in nitrogen supply (N1) significantly decreased the number of effective panicles and grains per panicle. Dry matter accumulation and translocation were the best under N2, with crop growth rates of 13.15 and 12.47 g·(m^-2^·d)⁻¹ for the two cultivars, respectively. Nitrogen-use-efficiency first increased and then decreased with increasing nitrogen applicatiopn rates. Under N2, nitrogen recovery efficiency exceeded 24%, and nitrogen agronomic efficiency of Byou 3611 reached 32.02 kg·kg⁻¹. Soluble sugar and sucrose contents, as well as the activities of sucrose phosphate synthase and sucrose synthase in grains, were highest under N2. Excessive nitrogen supply inhibited enzyme activity and sugar accumulation in grains. Significant genotypic differences were observed between the two cultivars: Byou 3611 performed better in terms of effective panicles, seed-setting rate, and dry matter translocation than Yixiangyou 2115.

**Discussion:**

This study indicates that Byou 3611 is superior to Yixiangyou 2115 in terms of grain yield and nitrogen-use-efficiency. The application rate of 150 kg·hm^-2^ nitrogen can simultaneously optimize yield components, dry matter translocation, nitrogen utilization and sugar metabolism. These findings provide a theoretical basis and technical support for nitrogen saving and high-efficiency rice cultivation in the hilly regions of Sichuan.

## 1 Introduction

Rice is one of the four major food crops in China and has one of the highest nitrogen demands.Optimal nitrogen fertilizer application is the most important cultivation measure to ensure a high and stable rice yield, and the application of nitrogen fertilizer directly affects the growth and development, yield and nitrogen-use-efficiency of rice (Neeraja et al., 2016; Yang et al., 2025; Zhang et al., 2025a). However, problems such as excessive application and unreasonable timing of nitrogen fertilizer application have long been prevalent in rice production in China. These issues not only cause a serious waste of fertilizer resources and a sharp decline in the nitrogen recovery efficiency and agronomic efficiency of rice but also trigger nitrogen loss from farmland, leading to agricultural non-point source pollution,including soil acidification and water eutrophication, which restricts the green and sustainable development of the rice industry (Wang et al., 2025; Liu et al., 2025). Therefore, promoting optimization of nitrogen fertilizer application and exploring the potential of rice varieties for efficient nitrogen utilization have become important approaches to achieve nitrogen conservation, efficiency increase, quality improvement and pollution reduction in rice production (Liu et al., 2025). Moreover, the combination of breeding nitrogen-efficient rice varieties and supporting reduced nitrogen application is a key measure to overcome the predicament of nitrogen fertilizer application from the perspective of coordination between genetic characteristics of varieties and cultivation measures (Devika et al., 2025).

The hilly area of Sichuan Province is a core production area in the southwest rice region of China and is the main rice-planting area in Sichuan Province. This region features a complex terrain, fragmented cultivated land, uneven soil fertility levels, a climate with high temperature and humidity, and an uneven temporal and spatial distribution of light. Its unique site conditions result in significant differences in the cultivation and management modes of rice production compared with rice production areas on plans (Xing and Wang, 2024). In recent years, with the advancement of agricultural modernization, nitrogen fertilizer input in rice production in the hilly areas of Sichuan has been continuously increasing. However, affected by extensive fertilization methods and the mismatch of nitrogen use characteristics of varieties, the nitrogen-use-efficiency of rice in this region is generally low, at about 30%, far lower than the national average level. At the same time, problems such as the decline in soil quality and the deterioration of farmland ecological environment caused by excessive nitrogen fertilizer application have become increasingly prominent, which has become the main bottleneck restricting the high-quality development of the rice industry in this region (Bao et al., 2018; Xu et al., 2025). Therefore, selecting nitrogen-efficient rice varieties and exploring the matching threshold of reduced nitrogen fertilizer application are of great practical significance for improving the nitrogen-use-efficiency of rice, reducing nitrogen fertilizer input and realizing green and high-yield cultivation in the hilly rice area of Sichuan Province.

Nitrogen-efficient rice varieties possess genetic characteristics such as strong nitrogen absorption capacity, efficient nitrogen distribution, and high assimilation efficiency. They can maintain a relatively high yield level under low-nitrogen conditions and achieve coordinated improvement in yield and nitrogen-use-efficiency under an appropriate nitrogen application rate (He et al., 2024; Zhang et al., 2025b). At present, a large number of studies have been conducted on the regulatory effects of reduced nitrogen fertilizer application on rice yield and nitrogen utilization and these have confirmed that an appropriate level of nitrogen fertilizer application can optimize nitrogen accumulation and translocation of rice plants, reduce unacceptable retention of nitrogen in vegetative organs, avoid the phenomenon of luxury nitrogen uptake, and promote the distribution of nitrogen to sink organs such as grains, thereby improving nitrogen-use-efficiency (Wichaidist et al.,2025; Sandeep et al.,2025). However, most existing studies have concentrated on rice areas on plains, such as the middle and lower reaches of the Yangtze River and Northeast China. Owing to the unique ecological conditions of the hilly area of Sichuan Province, the principles governing yield formation and nitrogen utilization of nitrogen-efficient rice varieties have not been clarified. In addition, there are significant genotypic differences in the responses of different nitrogen-efficient rice varieties to nitrogen fertilizer reduction, suitable nitrogen application thresholds and the synergistic mechanism of nitrogen utilization in hilly areas (Yang et al., 2011). Furthermore, the level of agricultural mechanization in rice production in the hilly area of Sichuan is low, with manual broadcasting being the main fertilization method. The operability and practicability of reducing nitrogen fertilizer application have become key to technological promotion. Research on reduced nitrogen application based on the main promoted nitrogen-efficient varieties in the local area can provide a scientific basis for formulating simple and efficient nitrogen fertilizer application schemes in the region.

Based on this, this study used two main rice varieties with significant differences in nitrogen utilization in the hilly area of Sichuan Province as experimental materials and set different nitrogen fertilizer reduction gradients in combination with the actual fertilization situation of rice production in the region. Core indicators, including rice yield components, plant nitrogen accumulation, nitrogen recovery efficiency, and nitrogen agronomic efficiency, were systematically determined. The objectives of this study were to explore the regulatory effects of reduced nitrogen fertilizer application on yield, nitrogen accumulation, and utilization of nitrogen-efficient rice varieties in the hilly area of Sichuan Province, to clarify the optimal rates of nitrogen fertilizer application, and to reveal differences in the responses of rice varieties with different nitrogen efficiencies to variation in rates of nitrogen fertilizer application. This research is expected to provide theoretical support and practical schemes for the determinnation of an supporting nitrogen fertilizer reduction technology system for nitrogen-efficient rice varieties in Sichuan Province and serve as a reference for the popularization and application of nitrogen-saving and efficiency-increasing rice cultivation technologies in the southwest hilly rice region.

## 2 Materials and methods

### 2.1 Experimental sites

**Figure 1:**
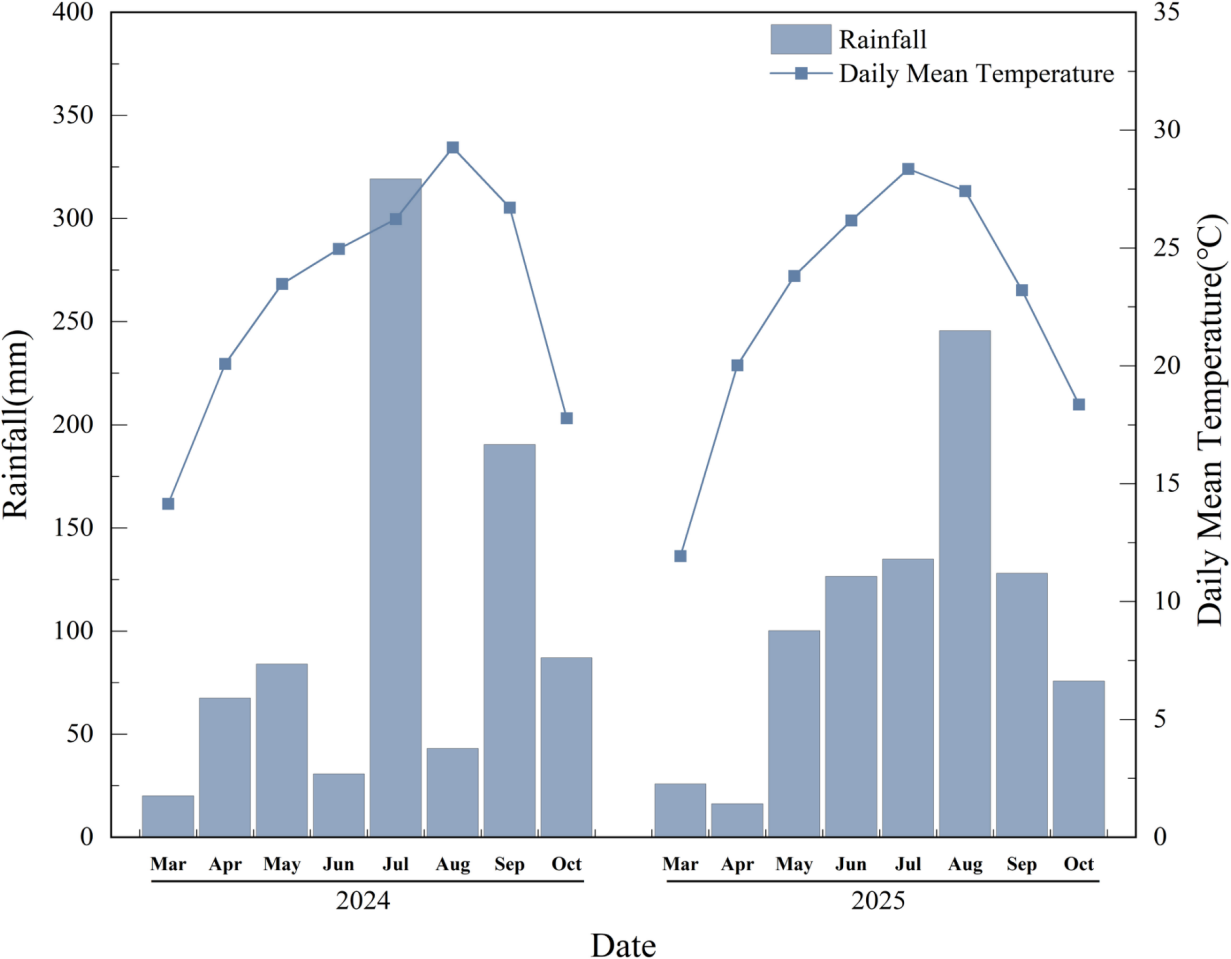
Meteorological data of the experimental area, including temperature and rainfall in 2024–2025.

Field experiments were conducted from 2024 to 2025 at the Agricultural Experimental Station of Southwest University of Science and Technology (31°53′ N, 104°70′ E). The soil was fallowed in winter and had medium-to-high fertility. The basic physicochemical properties of the plough layer (0–20 cm) were as follows: total nitrogen 1.48 g kg^-^¹, total phosphorus 0.83 g kg^-^¹, total potassium 1.65 g kg^-^¹, available phosphorus 18.64 mg kg^-^¹, available potassium 132.18 mg kg^-^¹, and pH 7.0. Meteorological conditions (temperature and rainfall) for 2024 and 2025 are shown in **Figure 1**.

### 2.2 Experimental design

A split-plot design was employed. Two rice varieties were used: Byou 3611(BY3611) and Yixiangyou 2115 (YXY2115).In this study, BY3611, a rice line with high nitrogen use efficiency independently developed by the Southwest University of Science and Technology, and the control line YXY2115 were selected. These two lines exhibited significant differences in nitrogen-use-efficiencies while sharing consistent growth duration and regional adaptability. This allowed for the effective control of environmental errors, formed a typical comparative gradient,and enabled accurate dissection of the physiological mechanisms underlying high nitrogen-use-efficiency in rice, ensuring both research uniqueness and experimental comparability.Four nitrogen (N) application rates were set as subplots:180 kg N hm^-^² (N3) 150 kg N hm^-^² (N2) 120 kg N hm^-^² (N1) and 0 kg N hm^-^² (N0). Each plot was 60 m² and was applied in triplicate. Rice seedlings were raised on April 20 and transplanted manually on May 22,with a spacing of 16 × 33 cm in both years. Harvesting was performed on September 4. Urea (46% N) was used as nitrogen fertilizer, calcium superphosphate (12.5% P_2_O_5_) as phosphorus fertilizer, and potassium chloride (60% KCl) as potassium fertilizer. Nitrogen was applied at a ratio of 3:3:4 (basal: tillering: panicle). Phosphorus (90 kg P_2_O_5_ hm^-^²) and potassium (180 kg KCl hm^-^²) were applied as basal fertilizers one day before transplanting. Disease, pest, and weed management followed the local high-yielding field practices.

### 2.3 Measurements and calculations

#### 2.3.1 Dry matter accumulation and leaf area

At the heading and maturity stages, five representative hills were sampled based on the average tiller number. Plants were separated into stem sheaths, leaves, and panicles, fixed at 105 °C for 30 min, and dried to a constant weight at 75 °C.

Leaf area was measured using the specific leaf weight method. Twenty leaves were randomly selected per plant, and a 15-cm segment was cut from each leaf as the standard sample. The total width (w, m) of 25 standard leaves was measured. After drying, the dry weights of standard and total leaves were recorded.Lear area per plant was calculated using the following formula:

Leaf area per plant = total leaf dry weight × 0.1 × w/Standard leaf dry weight.

The leaf area index (LAI) was calculated accordingly.

#### 2.3.2 Total nitrogen, soluble sugar and sucrose contents

Dried plant samples were ground and sieved (60-mesh) for total nitrogen(N), soluble sugar, and sucrose analyses. Total N was digested by the H_2_SO_4_-H_2_O_2_ method and determined using a FOSS 8400 semi-automatic Kjeldahl analyzer. Soluble sugar and sucrose in leaves and panicles at full heading were measured using anthrone colorimetry and resorcinol colorimetry, respectively.The following equations were applied:

Nitrogen accumulation (kg hm^-^²) = dry matter accumulation per unit area × N concentration.

N agronomic efficiency (NAE, kg kg^-^¹) = (Grain yield in N plot − Grain yield in N0 plot)/N application rate.

N recovery efficiency (NRE, %) = (Total N accumulation in N plot − Total N accumulation in N0 plot)/N application rate × 100.

#### 2.3.3 Enzyme activity assays

Samples were collected at 15 days after flowering. Three uniform representative hills were sampled in triplicate. Samples were separated into leaves and grains, frozen in liquid nitrogen, and stored at −80 °C.

#### 2.3.4 Grain yield and yield components

Rice was harvested separately from each plot at maturity.to determine yield. Five representative hills were collected to investigate effective panicles, filled grains per panicle, 1000-grain weight, and seed-setting rates.

### 2.4 Data analyses

Data were processed using Microsoft 365. Statistical analyses and analysis of variance (ANOVA) were performed using SPSS Statistics 23. Significant differences among treatments were tested using the least significant difference (LSD) method at *P* < 0.05. All figures were plotted using Origin 2022 software. Values are presented as means.

## 3 Results

### 3.1 Responses of grain yield and yield components to reduced nitrogen application

ANOVA results (**Table 1**) revealed that year (Y), variety (V), nitrogen application rate (N), and their interaction (V×N) significantly affected grain yield of both BY3611 and YXY2115 (*P* < 0.01). The three-factor Y × V × N interation showed no significant effects. Except for the grain number per panicle, which was not affected by year, Y, V, N, and V×N significantly influenced the number of effective panicles, seed-setting rate, and 1000-grain weight.

**Table 1:** Responses of rice yield and yield components to reduced nitrogen application.

Year	Variety	N rate	EP	GN	SS	TGW	RY
			(×10^4^·hm^−2^)	(panicle^−1^)	(%)	(g)	(kg·hm^-2^)
2024	BY3611	N3	214.02abc	223.84a	90.39cde	25.19h	10903.61d
		N2	215.30abc	219.20a	93.44a	26.17fg	11538.44c
		N1	215.50abc	208.38b	91.49bc	25.35gh	10415.19de
		N0	169.61fg	186.36c	89.55cde	25.21h	7136.49i
	YXY2115	N3	180.6def	171.65ef	87.14fg	36.46c	9853.19fg
		N2	203.60c	180.07d	85.78g	36.49c	11479.08c
		N1	185.81d	166.86fg	87.27fg	35.15d	9484.23g
		N0	158.01h	155.18h	83.63h	35.48d	7267.26i
2025	BY3611	N3	218.77a	223.83a	91.33bc	26.81f	11987.31bc
		N2	217.27ab	219.96a	94.55a	28.03e	12663.67a
		N1	217.35ab	213.08b	92.75ab	26.92f	11562.64c
		N0	171.58efg	187.78c	90.88bcd	26.85f	7864.04h
	YXY2115	N3	182.37de	173.17e	87.46fg	38.12a	10529.40de
		N2	205.52bc	181.87cd	88.61ef	37.45ab	12403.31ab
		N1	190.76d	165.04g	89.07def	36.84bc	10320.72ef
		N0	162.96gh	157.29h	85.52g	36.52c	8011.97h
F value		Y	35.50**	1.64	21.40**	103.94**	118.73**
		V	128.75**	1647.30**	258.58**	4811.39**	50.15**
		N	111.85**	163.36**	21.04**	10.96**	512.24**
		V×N	10.69**	18.50**	5.90**	5.57**	16.84**
		Y×V×N	0.15	0.87	0.59	0.71	0.32

BY3611, B you 3611; YXY2115, Yixiangyou2115; N3, 180kg/hm2; N2, 150kg/hm2; N1, 120kg/hm2; N0, 0kg/hm2; EP, Effective Panicles; GN, Grain Number; SS, Seed Setting Rate; TGW, Thousand Grain Weight; RY, Rice Yield; Different lowercase letters in the same column represent ANOVA results between different treatments in the same year; Y, V and N in the F value are year, variety and nitrogen fertilizer treatment, respectively. V × N and Y ×V×N are the interaction between year, variety and nitrogen fertilizer treatment, respectively. The asterisk indicates significant difference, *P < 0.05, **P < 0.01.

Nitrogen application rate significantly regulated yield and yield components of both varieties. All N treatments significantly increased the number of effective panicles, grain number, seed-setting rate, and yield compared with N0. N2 performed best and achieved the highest yield in both years. In 2024, the yields of BY3611 and YXY2115 under N2 were 11538.44 kg·hm^-^² and 11479.08 kg·hm^-^², respectively, and further increased to 12663.67 kg·hm^-^² and 12403.31 kg·hm^-^² in 2025. When N was reduced to N1, the yields of both varieties decreased. Moreover, N3 failed to boost yield and slightly reduced seed-setting rate, indicating that excessive N was not optimal for yield improvement. Under N0, effective panicles and grain number decreased sharply at a low seed-setting rate, suggesting that an appropriate N supply was essential for the high yields of these two N-efficient varieties. Significant genotypic differences were observed in yield components. BY3611 exhibited significantly larger effective panicles, grain number, and seed-setting rates than YXY2115, constituting its core yield advantage. In contrast, YXY2115 had a prominent 1000-grain weight (>35 g), that way significantly higher than that of BY3611 (25–28 g), and served as the main yield contributor of YXY2115. N2 significantly increased the number of effective panicles and optimized the seed-setting rate in both varieties. The increased number of effective panicles accounted for the substantial yield gain of YXY2115 under N2, whereas BY3611 achieved a stable yield improvement via a high seed-setting rate. The Yield and yield components of both varieties showed consistent trends across the two years; values in 2025 were slightly higher than in 2024, but the interaction pattern between variety and N rate remained unchanged, further verifying that N2 was the optimal N rate for high yields of BY3611 and YXY2115 in hilly regions of Sichuan.

### 3.2 Effects of reduced nitrogen application on dry matter accumulation and translocation

As shown in **Table 2**, Y, V, N, and V×N significantly affected dry matter accumulation, translocation, and crop growth rate (CGR) of both varieties (*P* < 0.01). The three-factor interaction Y×V×N also significantly regulated dry matter accumulation at maturity and apparent translocation amount, except for CGR, which was not significantly affected by variety or the three-factor interaction,demonstrating that the rate of N application was the dominant factor regulating dry matter metabolism, with significant genotypic variations. The N application rate significantly modulated dry matter accumulation, and N2 was optimal for dry matter accumulation and translocation. Dry matter accumulation at maturity and CGR under N2 were the highest in both years; in 2025, CGR of BY3611 and YXY2115 exhibited 13.72 and 13.09 g·(m²·d)^-^¹, respectively. Increasing N to N3 enhanced dry matter accumulation at full heading, but did not significantly improve dry matter at maturity or reduce dry matter translocation efficiency. Reducing N supply to N1 or N0 significantly decreased dry matter at both heading and maturity, and sharply lowered the CGR. Dry matter accumulation and CGR in 2025 were higher than those in 2024, reflecting the positive effects of annual climatic conditions. Significant genotypic differences were observed in dry matter accumulation and translocation. BY3611 exhibited stronger dry matter translocation than YXY2115; dry matter translocation and apparent translocation of BY3611 were significantly higher under all N treatments, especially under N2 (2127.52 kg·hm^-^², the highest among all treatments) with suitable apparent translocation efficiency. Conversely, YXY2115 showed superiority in post-anthesis dry matter accumulation; its dry matter at maturity under N2 in 2025 was reached 15596.77 kg·hm^-^² (peak in two years), providing a key material basis for yield formation.

**Table 2:** Effects of reduced nitrogen application on dry matter accumulation and translocation in rice.

Year	Variety	N rate	DMFH (kg/hm^2^)	DMM(kg/hm^2^)	DMT (kg/hm^2^)	ATA (kg/hm^2^)	ATE (%)	CGR(g/(m^2^·d))
2024	BY3611	N3	11758.01c	14173.32f	1644.87b	1435.67b	22.49a	9.02e
		N2	11277.52d	14405.57e	2112.47a	1021.5d	17.13cd	12.49bc
		N1	9765.50g	13183.21h	1277.73cd	784.23e	15.76de	10.4d
		N0	8520.23i	10976.03l	1283.57cd	736.17e	18.15c	7.01f
	YXY2115	N3	10951.50e	13711.63g	1371.87cd	1074.13d	18.64c	10.32d
		N2	9826.57g	14167.07f	1019.43ef	624.83f	11.79f	11.86c
		N1	8625.23i	12681.13j	847.47fg	253.73g	5.93h	10.28d
		N0	7377.83k	10405.70m	846.10fg	341.46g	9.97g	7.13f
2025	BY3611	N3	12694.17a	15337.8b	1659.73b	1654.90a	23.41a	10.25d
		N2	12103.43b	15126.43c	2142.57a	1188.43c	18.10c	13.72a
		N1	10677.43f	14436.30e	1116.73de	576.13f	10.18fg	11.63c
		N0	9475.67h	11908.52k	1351.37cd	991.10d	20.73b	8.23e
	YXY2115	N3	11796.36c	14698.27d	639.87g	358.23g	5.59h	11.55c
		N2	10935.18e	15596.77a	1276.67cd	845.03e	14.21e	13.09ab
		N1	9403.03h	13038.73i	1380.27c	779.03e	15.92de	11.51c
		N0	8294.47j	11080.33l	1151.23cde	596.43f	14.50e	8.36e
F value		Y	725.46**	1629.09**	1.61	21.86**	1.33	46.52**
		V	1124.84**	500.32**	168.22**	524.65**	431.85**	0.91
		N	1979.85**	5235.55**	33.66**	162.15**	62.12**	137.74**
		V×N	8.50**	95.59**	24.95**	53.13**	39.73**	5.15**
		Y×V×N	1.96	50.16**	15.06**	79.80**	102.95**	0.96

BY3611, B you 3611; YXY2115, Yixiangyou2115; N3, 180kg/hm2; N2, 150kg/hm2; N1, 120kg/hm2; N0, 0kg/hm2; DMFH, Dry Matter at Full Heading; DMM, Dry Matter at Maturity; DMT, Dry Matter Translocation; ATA, Apparent Translocation Amount; ATE, Apparent Translocation Efficiency; CGR, Crop Growth Rate. Different lowercase letters in the same column represent ANOVA results between different treatments in the same year; Y, V and N in the F value are year,variety and nitrogen fertilizer treatment, respectively. V × N and Y ×V×N are the interaction between year, variety and nitrogen fertilizer treatment, respectively. The asterisk indicates significant difference, *P < 0.05, **P < 0.01.

Interactive effects of the N application rate and variety on dry matter translocation were detected.N2 significantly increased dry matter translocation of BY3611 and optimized post-anthesis dry matter accumulation of YXY2115. Under N3, apparent translocation efficiency increased but dry matter translocation did not rise synchronously, showing high pre-anthesis accumulation but low post-anthesis translocation. CGR was consistent with dry matter accumulation, with the highest values under N2 and no significant differences between varieties, suggesting that appropriate N reduction narrowed the gap in growth rate and facilitated efficient dry matter accumulation.

### 3.3 Effects of reduced nitrogen application on leaf area

**Figure 2:**
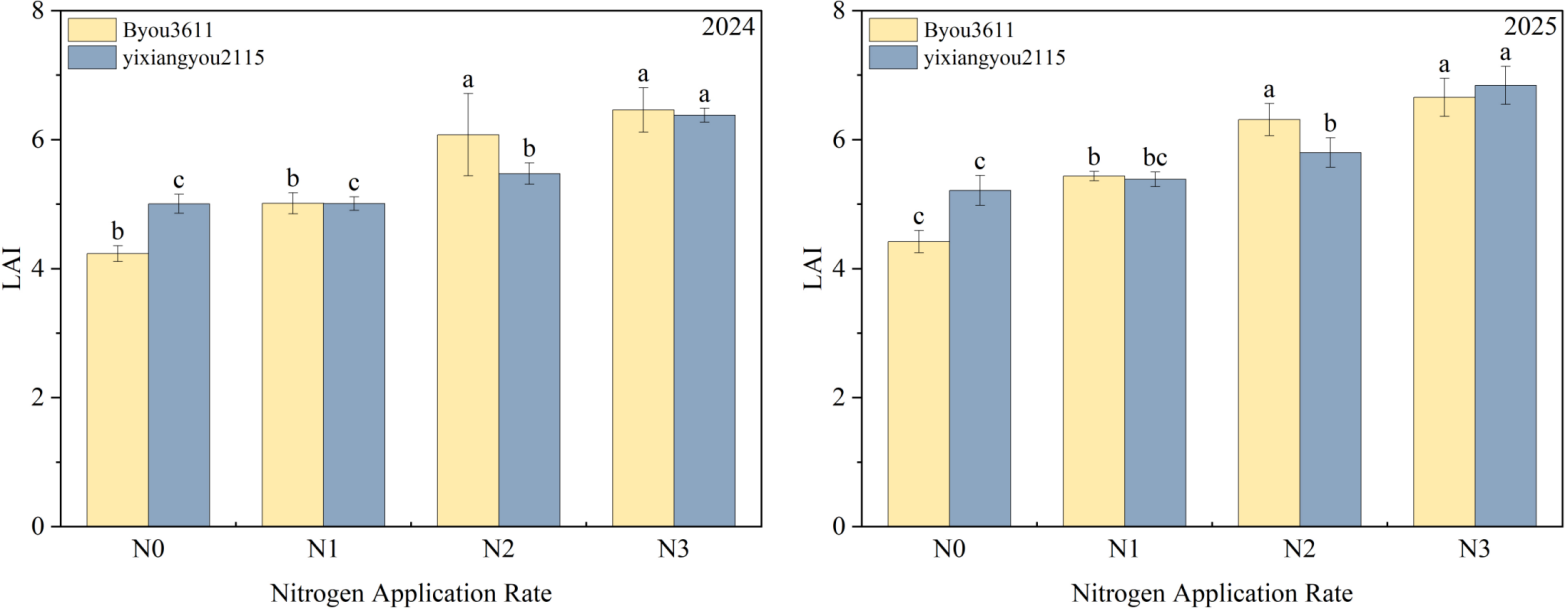
Effects of reduced nitrogen application on leaf area. N3:180kg/hm2;N2:150kg/hm2;N1:120kg/hm2;N0:0kg/hm2; DMFH, Dry Matter at Full Heading; DMM, Dry Matter at Maturity; DMT, Dry Matter Translocation; ATA, Apparent Translocation Amount; ATE, Apparent Translocation Efficiency; CGR, Crop Growth Rate. Different lowercase letters in the same column represent ANOVA results between different treatments in the same year.

ANOVA (**Figure 2**) demonstrated that N application rate, variety, year and V×N interaction significantly regulated leaf area.The leaf area in 2025 was generally higher than that in 2024, and BY3611 had a larger leaf area than YXY2115, with obvious gradient changes along the N rates.

Leaf area increased with increasing N application rates. and leaf area in 2025 was 5.53% higher thanthat in 2024. Compared to N2, N1, and N0, N3 significantly increased the leaf area by 11.33%, 26.29% and 40.0%, respectively. In summary, appropriate N supply significantly promoted leaf area expansion. In 2025,the environment was more favorable for leaf area formation, and BY3611 a showed stronger N response with a maximum leaf area of 6.46, indicating greater dry matter production potential than YXY2115.

### 3.4 Effects of reduced nitrogen application on nitrogen accumulation and utilization

ANOVA (**Table 3**) showed that Y, V, and N significantly affected N accumulation at full heading and maturity in both varieties (*P* < 0.01). The N application rate had an extremely significant regulatory effect on N recovery efficiency (NRE) and N agronomic efficiency (NAE). The V×N interaction significantly affected only NAE, whereas Y×V×N had no significant effect on any index. Rate of N rate application was the core factor regulating N accumulation and utilization, with significant genotypic differences in N use efficiency (NUE).

**Table 3:** Effects of reduced nitrogen application on nitrogen accumulation and utilization in rice.

Year	Variety	N rate	NAFH(kg/hm2)	NAM(kg/hm2)	NRE(%)	NAE(kg/kg)
2024	BY3611	N3	119.01bcd	139.27ef	16.59e	20.93cd
		N2	111.77fg	146.52bcd	24.73a	29.35ab
		N1	106.42h	133.90g	20.42cd	27.32b
		N0	51.80j	58.83i	/	/
	YXY2115	N3	121.30ab	143.67cd	16.93e	14.37e
		N2	115.22ef	148.80ab	23.73ab	28.08ab
		N1	111.50g	139.01ef	21.50bc	18.48d
		N0	55.72i	63.20h	/	/
2025	BY3611	N3	122.73ab	143.01de	16.43e	22.91c
		N2	115.51de	150.23ab	24.52a	32.02a
		N1	109.96g	137.46fg	20.03cd	30.82ab
		N0	53.33ij	63.43h	/	/
	YXY2115	N3	124.57a	147.57bc	18.01de	13.99e
		N2	119.52bc	152.07a	25.13a	29.28ab
		N1	115.93cde	142.97de	23.83ab	19.24cd
		N0	55.97i	64.37h	/	/
F value		Y	26.40**	27.67**	1.84	4.46*
		V	36.63**	30.01**	4.64*	75.38**
		N	2689.23**	3795.95**	77.97**	76.82**
		V×N	1.41	1.29	2.36	10.10**
		Y×V×N	0.16	0.45	0.22	0.06

BY3611, B you 3611; YXY2115, Yixiangyou2115; N3, 180kg/hm2; N2, 150kg/hm2; N1, 120kg/hm2; N0, 0kg/hm2; NAFH, Nitrogen Accumulation at Full Heading; NAM, Nitrogen Accumulation at Maturity; NRE, nitrogen recovery efficiency; NAE, nitrogen agronomy efficiency. Different lowercase letters in the same column represent ANOVA results between different treatments in the same year; Y, V and N in the F value are year,variety and nitrogen fertilizer treatment, respectively. V × N and Y ×V×N are the interaction between year, variety and nitrogen fertilizer treatment, respectively. The asterisk indicates significant difference, *P < 0.05, **P < 0.01.

The N application rate significantly regulated N accumulation. N-treated plots had significantly higher N accumulation at heading and maturity than N0, showing a trend of increasing first and then stabilizing with rising N rate. N3 produced the highest N accumulation at heading but failed to significantly improve N accumulation at maturity. N2 maintained high N accumulation at maturity for both varieties; in 2025, values of YXY2115 and BY3611 reached 152.07 and 150.23 kg·hm^-^², respectively, suggesting that appropriate N reduction satisfied late-stage N demand, whereas excessive N caused redundant pre-anthesis accumulation and reduced late-stage utilization efficiency.

NUE first increased and then decreased with a teduction in the N application rate, and N2 was optimal. NRE and NAE under N2 were the highest in both years. In 2025, NRE of YXY2115 and BY3611 were 25.13% and 24.52%, respectively; maximum NAE of BY3611 was 32.02 kg·kg^-^¹, showing excellent NUE. N3 significantly reduced NRE and NAE, indicating severe N waste under excessive N. N1 ranked second in NUE, slightly lower than N2 but significantly higher than N3.

Genotypic differences were significant: YXY2115 had slightly higher N accumulation at heading and maturity than BY3611, whereas BY3611 exhibited a significantly higher NAE. The NRE of both varieties under N2 exceeded 24% with no significant difference. N accumulation and and NAE in 2025 were slightly higher than those in 2024, showing a minor positive environmental effect without altering the regulatory pattern and further confirming that N2 is suitable for efficient N accumulation and utilization in both varieties.

### 3.5 Effects of reduced nitrogen application on sucrose and soluble sugar contents

ANOVA (**Table 4**) revealed that N application rate significantly affected soluble sugar and sucrose contents in leaves and grains of both varieties (*P* < 0.01). The V×N interaction significantly affected leaf soluble sugar, whereas Y×V×N had no significant effect on sugar content, indicating that the rate of application of N was the core factor regulating sugar accumulation.

**Table 4:** Effects of reduced nitrogen application on sucrose and soluble sugar contents in rice.

Year	Variety	N rate	SS(mg/g DW)		Suc(mg/g DW)	
			Leaf	Grain	Leaf	Grain
2024	BY3611	N3	178.22a	36.87cd	138.33a	37.2b
		N2	161.71b	41.60a	121.82b	41.17a
		N1	149.32c	35.81de	109.42cd	26.33c
		N0	139.80d	31.53ij	99.90e	20.52de
	YXY2115	N3	174.38a	34.22efg	134.41a	36.03b
		N2	158.70b	38.70b	118.80b	40.51a
		N1	149.61c	32.62ghi	109.72cd	26.07c
		N0	142.30d	29.81j	102.41e	18.42e
2025	BY3611	N3	179.34a	37.87bc	139.23a	37.17b
		N2	163.47b	42.80a	123.47b	41.84a
		N1	150.72c	36.82cd	111.47c	26.91c
		N0	141.27d	32.43hi	104.73de	22.55d
	YXY2115	N3	175.57a	35.13def	134.97a	37.26b
		N2	158.87b	39.43b	119.94b	42.43a
		N1	152.47c	34.01fgh	113.27c	26.53c
		N0	143.81d	30.90ij	104.47de	19.46de
F value		Y	3.26	13.32**	4.71*	3.32
		V	1.62	82.98**	1.84	2.38
		N	355.50**	187.93**	229.17**	311.10**
		V×N	4.22*	1.48	2.05	1.12
		Y×V×N	0.15	0.11	0.19	0.22

BY3611, B you 3611; YXY2115, Yixiangyou2115; N3, 180kg/hm2; N2, 150kg/hm2; N1, 120kg/hm2; N0, 0kg/hm2; SS, Soluble Sugar; Suc, Sucrose. Different lowercase letters in the same column represent ANOVA results between different treatments in the same year; Y, V and N in the F value are year,variety and nitrogen fertilizer treatment, respectively. V × N and Y ×V×N are the interaction between year, variety and nitrogen fertilizer treatment, respectively. The asterisk indicates significant difference, *P < 0.05, **P < 0.01.

The N application rate regulated sugar content in a consistent pattern for both varieties. Leaf soluble sugar and sucrose levels increased significantly with increasing N application rate, with the highest values under N3, followed by N2 and N0, showing the same trend over the two years. This suggests that appropriate N promoted carbohydrate synthesis and accumulation in leaves, providing material for grain-filling. However, grain soluble sugar and sucrose contents first increased and then decreased with increasing N application rates, and N2 was optimal for grain sugar accumulation. In 2025, maximum soluble sugar and sucrose in BY3611 grains under N2 was 42.80 and 41.84 mg·g^-^¹ DW, respectively and 39.43 and 42.43 mg·g^-^¹ DW for YXY2115. Grain sugar content decreased significantly under N3, indicating that excessive N supply inhibited translocation and accumulation of photosynthates from leaves to grains.

Obvious genotypic differences existed in sugar accumulation: BY3611 had significantly higher grain soluble sugar than YXY2115, a key quality advantage, whereas no significant differences were detected in leaf sugar or grain sucrose. The N application rate showed no obvious interactive effect on sugar accumulation, except for leaf soluble sugar, and trends were highly consistent between varieties, indicating the universal regulation of N reduction on sugar accumulation.

The amount of soluble sugars in 2025 was slightly higher than that in 2024, but the core regulatory patterns remain unchanged. Under N0, the sugar content in leaves and grains was the lowest, demonstrating that N deficiency significantly inhibited photosynthate synthesis and translocation. N2 balanced leaf sugar synthesis and efficient translocation to grains, achieving synergistic optimization of source supply and sink accumulation.

### 3.6 Effects of reduced nitrogen application on sucrose phosphate synthase and sucrose synthase activities

Two-year experiments (**Table 5**) indicated that N application rate significantly affected sucrose phosphate synthase (SPS) and sucrose synthase (SuSy) activities in leaves and grains of both varieties (*P* < 0.01). Variety significantly affected leaf SPS and SuSy and significantly affected grain SPS, but had no significant effect on grain SuSy. The V×N interaction extremely significantly or significantly affected grain SPS, leaf SuSy, and grain SuSy, but not leaf SPS. The three-factor interaction significantly affected leaf and grain SuSy. These results demonstrated that key enzymes in sucrose metabolism were regulated by multiple factors, with rate of N rate application the dominant factor and a significant V×N interaction. Leaf SPS activity decreased significantly with declining rates of N supply; N3 showed the highest leaf SPS in both years (44.82 and 44.63 mg·source^-^¹·g^-^¹ FW for BY3611; 40.62 and 41.67 mg·source^-^¹·g^-^¹ FW for YXY2115), and N0 the lowest, indicating that N supply promoted leaf SPS and sucrose synthesis. However, grain SPS and leaf/grain SuSy activity first increased and then decreased with increasing N application rates, and N2 was optimal for enzyme expression. Grain SPS under N2 was significantly higher than other treatments; in 2025, maximum values of BY3611 and YXY2115 were 31.91 and 28.72 mg·source^-^¹·g^-^¹ FW, respectively. Leaf and grain SuSy activities under N2 were also high, providing enzymatic support for sucrose translocation and accumulation. Grain SPS and SuSy activities decreased under N3, suggesting that excessive supply of N enhanced leaf sucrose-synthesizing enzymes but inhibited grain sucrose-metabolizing enzymes, which was unfavorable for photosynthate translocation. All enzyme activities were the lowest under N0, indicating that N deficiency significantly suppressed key enzymes and reduced sucrose synthesis and translocation efficiency. Significant genotypic differences were observed in enzyme activities: BY3611 had significantly higher leaf SPS and SuSy than YXY2115, a core advantage in leaf sucrose synthesis. YXY2115 showed superiority in grain SuSy under N2 (42.63 and 46.57 mg·source^-^¹·g^-^¹ FW in 2024 and 2025, the highest in each year), providing an enzymatic basis for grain sucrose accumulation. The V×N interaction significantly regulated enzyme activities; and N2 synergistically optimized the leaf and grain sucrose-metabolizing enzymes of both varieties, achieving enzymatic matching between leaf synthesis and grain accumulation.

**Table 5:** Effects of reduced nitrogen application on sucrose phosphate synthase and sucrose synthase activities in rice.

Year	Variety	N rate	SPS(mg/source/g FW)		SuSy(mg/source/g FW)	
			Leaf	Grain	Leaf	Grain
2024	BY3611	N3	44.82a	25.333cdef	55.57a	40.43ef
		N2	39.87bcd	29.62ab	50.43bc	44.83ab
		N1	35.91ef	22.80fghi	46.73de	38.41f
		N0	30.60hi	20.92hij	41.61gh	29.82i
	YXY2115	N3	40.62b	24.61cdefg	46.23def	35.47g
		N2	36.70ef	27.20bc	52.13b	42.63cd
		N1	33.12gh	23.83defg	43.63efgh	38.40f
		N0	29.58i	18.72j	40.41h	28.62i
2025	BY3611	N3	44.63a	27.17bc	50.13bc	43.60bc
		N2	40.17bc	31.91a	55.23a	40.92de
		N1	37.37de	23.27efgh	48.52cd	39.93ef
		N0	32.23ghi	22.40ghi	43.37fgh	32.21h
	YXY2115	N3	41.67b	25.97cd	48.21cd	41.23de
		N2	37.71cde	28.72b	51.47bc	46.57a
		N1	34.17fg	25.47cde	44.57efg	39.67ef
		N0	30.33hi	20.53ij	41.55gh	32.83h
F value		Y	4.21*	14.73**	2.25	48.20**
		V	37.71**	6.60*	31.59**	3.15
		N	138.19**	80.23**	80.43**	271.24**
		V×N	0.98	5.56**	4.07*	11.55**
		Y×V×N	0.27	0.31	6.59**	6.83**

BY3611:B you 3611;YXY2115:Yixiangyou2115; N3:180kg/hm2;N2:150kg/hm2;N1:120kg/hm2;N0:0kg/hm2; SPS: Sucrose Phosphate Synthase; SuSy: Sucrose Synthase. Different lowercase letters in the same column represent ANOVA results between different treatments in the same year; Y, V and N in the F value are year,variety and nitrogen fertilizer treatment, respectively. V × N and Y ×V×N are the interaction between year, variety and nitrogen fertilizer treatment, respectively. The asterisk indicates significant difference, *P < 0.05, **P < 0.01.

Grain SPS and SuSy activities in 2025 were slightly higher than those in 2024, indicating a minor positive environmental effect without altering the regulatory pattern or genotypic differences. Overall, plants within the N2 group exhibited optimized leaf and grain sucrose-metabolizing enzymes, ensuring efficient leaf sucrose synthesis and enhanced grain sucrose metabolism, laying a critical enzymatic foundation for photosynthate translocation and accumulation, which also explained the optimal grain sugar and yield under N2.

### 3.7 Correlation analysis among rice traits under reduced nitrogen application

**Figure 3:**
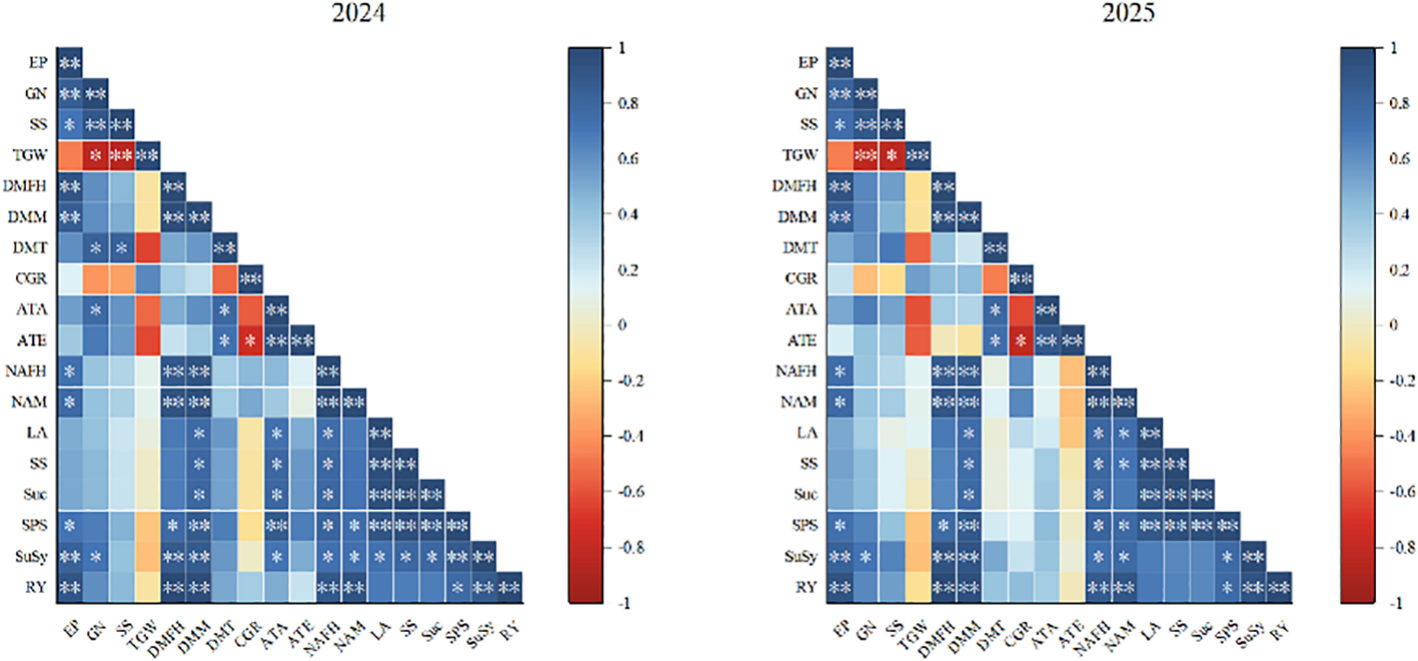
Correlation analysis among rice traits under reduced nitrogen application. EP, Effective Panicles; GN, Grain Number; SS, Seed Setting Rate; TGW, Thousand Grain Weight; DMFH, Dry Matter at Full Heading; DMM, Dry Matter at Maturity; DMT, Dry Matter Translocation; ATA, Apparent Translocation Amount; ATE, Apparent Translocation Efficiency; CGR, Crop Growth Rate; NAFH, Nitrogen Accumulation at Full Heading; NAM, Nitrogen Accumulation at Maturity; SS, Soluble Sugar; Suc, Sucrose; SPS, Sucrose Phosphate Synthase; SuSy, Sucrose Synthase; RY, Rice Yield. Different lowercase letters in the same column represent ANOVA results between different treatments in the same year; Y, V and N in the F value are year,variety and nitrogen fertilizer treatment, respectively. V × N and Y ×V×N are the interaction between year, variety and nitrogen fertilizer treatment, respectively. The asterisk indicates significant difference, *P < 0.05, **P < 0.01.

Correlation analyses of yield, dry matter, N utilization, sugar content, enzyme activity and leaf area revealed significant intrinsic relationships (**Figure 3**). Grain yield was positively correlated with most agronomic and physiological traits, to varying degrees. Yield was significantly and positively correlated with dry matter at maturity, NAE, grain soluble sugar, grain sucrose, grain SPS, grain SuSy, and leaf area but weakly or negatively correlated with leaf sugar and leaf enzymes. This indicated that grain sugar accumulation, sucrose-metabolizing enzymes, efficient dry matter and N utilization were core drivers of yield improvement, and that moderate leaf area expansion laid the foundation for yield formation. Dry matter accumulation was significantly and positively correlated with N accumulation and leaf area, and significantly correlated with NRE and NAE, suggesting that efficient N uptake was a prerequisite for dry matter accumulation, and leaf area expansion provided photosynthetic support. Sucrose-metabolizing enzymes and sugar contents showed obvious source-sink synergy: leaf SPS and SuSy were significantly positively correlated with leaf sugar, and grain enzymes with grain sugar;which showed a much stronger correlation with yield than leaf enzymes. In addition, NUE was significantly and positively correlated with grain sugar and grain enzymes. Correlations among traits were significantly weakened under excessive N, indicating that appropriate N (N2) promoted the synergistic improvement of traits, whereas excessive N supply disrupted source-sink translocation and metabolic balance.

## 4 Discussion

### 4.1 Effects of reduced nitrogen application on rice yield and dry matter production

Nitrogen (N) is a key factor in determining rice yield and dry matter accumulation, and N management is among the most important cultivation practices in rice production (Redda et al., 2022). N application mainly regulates yield components; increasing effective panicles is critical for yield improvement (Xie et al., 2019), and a reasonable N supply favors effective tillering and rational population construction (Hu et al., 2020). Increasing the N application rate within an appropriate range increases rice yield, but excessive N reduces it (Zhao et al., 2023). In this study, 150 kg N hm^-^² (N2) was the optimal rate for high yield of Byou 3611 and Yixiangyou 2115, which is consistent with previous findings (Zhang et al., 2018).

A prerequisite for yield improvement is the expansion of the effective sink size (Wang et al., 2023), which can be achieved by increasing the number of effective panicles and grains per panicle (Xue et al., 2011). In the present study, excessive N (180 kg hm^-^², N3) did not increase yield but slightly reduced seed-setting rate, indicating a threshold response of rice yield to N. Beyond this threshold, luxury N absorption occurs and the marginal yield benefit declines. The regulatory effect of rate of application of N on yield was achieved by synergistically optimizing yield components. N2 significantly increased the number of effective panicles and optimized the seed-setting rate, which was the main reason for yield enhancement. Excessive N reduction (N1, 120 kg hm^-^²) decreased effective panicles and grain number, failing to meet the population structure required for high yield,as previously observed (Liang et al.,2021). Byou 3611 achieved a significantly higher grain yield in the Chengdu Plain than in the hilly regions of Sichuan. Nevertheless, this variety demonstrates wide adaptability and stable yield, with increased production in both areas, making it suitable for cultivation in both the flat dams and hilly ecological zones of the southwest rice region.

Byou 3611 achieved a high yield mainly via more effective panicles, higher grain number and seed-setting rate, whereas Yixiangyou 2115 was characterized by a larger 1000-grain weight (>35 g), which was significantly higher than that of Byou 3611, consistent with previous reports (Qin et al.,2025). Notably, N2 synergistically optimized yield components of both varieties: it greatly increased the number of effective panicles in Yixiangyou 2115 and maintained a high seed-setting rate in Byou 3611, realizing complementary advantages. This confirms that appropriate declines in N supply narrows the gap in yield formation among N−efficient varieties and achieves synchronous high yield. Dry matter accumulation is a key determinant of rice yield (Li et al., 2025). As a core regulator of dry matter metabolism, rate of N supply interacts with genetic characteristics to modulate dry matter accumulation, translocation and crop growth rate (CGR) (Yang and Zhu, 2020). In this study, N2 was found to be optimal for dry matter production. Dry matter at maturity and CGR under N2 were the highest in both years; in 2025,maximum CGR was 13.72 and 13.09 g·(m²·d)^-^¹ for Byou 3611 and Yixiangyou 2115, respectively. Excessive supply of N (N3) increased dry matter at heading but reduced translocation efficiency, showing a pattern of high pre-anthesis accumulation but low post-anthesis translocation. Severe reductions in supply of (N1, N0) significantly decreased dry matter accumulation at both stages and sharply reduced the CGR, consistent with those of previous studies (Liu et al., 2020), which reported that dry matter translocation efficiency after heading under conventional N was lower than that under 20% N reduction, leading to the decoupling of dry matter production from yield formation. Excessive N retention in vegetative organs impedes N translocation from stems, sheaths, and leaves to grains, which is the main cause of reduced dry matter translocation efficiency (Yang and Zhu, 2020). Poshtmasari et al., 2007 demonstrated that post-graining dry matter accumulation was closely associated with the stay-green trait and that dry matter remobilization capacity of different varieties was positively correlated with the sink capacity of their panicles. Among these, the Tarom variety, although lacking remobilization capacity, exhibited strong sink characteristics. The high seed-setting rate of Byou 3611 corresponded to strong dry matter translocation, and the large-grain type of Yixiangyou 2115 required continuous post-anthesis dry matter supply, reflecting a source–sink synergistic mechanism for yield formation. Appropriate reductions of N supply optimized photosynthetic system development and photosynthate translocation by regulating N uptake and distribution, homogenized growth rate among N−efficient varieties, and promoted efficient dry matter production. In addition, annual climatic conditions positively affected dry matter accumulation; values in 2025 were higher than those in 2024, owing to more suitable temperature and solar radiation distribution for photosynthesis (Zhang et al., 2018). Peng et al. (Peng et al., 2019) also reported that favorable climate enhanced CGR and dry matter accumulation, indicating that dry matter metabolism is co-regulated by N and environment, with N as the dominant factor.

### 4.2 Effects of reduced nitrogen application on nitrogen accumulation and use efficiency

N uptake and utilization in rice depend closely on genotype and rate of N supply (Wang et al., 2023). In the present study, N2 simultaneously improved N accumulation and N-use-efficiency, effectively alleviating the low N-use-efficiency (NUE) caused by excessive N in the hilly region of Sichuan (Liang et al., 2021). In 2025, N recovery efficiency (NRE) of both varieties under N2 exceeded 24%, and maximum N agronomic efficiency (NAE) of Byou 3611 was 32.02 kg·kg^-^¹, consistent with Shi (Shi et al., 2020), who reported that 20%–40% N reduction combined with green manure significantly increased N uptake and NUE, with the best agronomic benefit at 20% N reduction, highly consistent with our findings (Sun et al., 2012).

N accumulation first increased and then stabilized with increasing N application rate. N3 produced the highest N accumulation at heading but failed to synchronously improve N accumulation at maturity. This may be because excessive supply of N causes redundant uptake at early growth stages, large N retention in vegetative organs, and reduced N assimilation and translocation to grains at later stages (Zhu et al., 2020). NUE decreased significantly under N3, mainly because excessive N supply exceeded the uptake threshold of N−efficient rice, and unused N was lost via leaching and volatilization (Tao et al., 2026). In China, the efficiency of N fertilizer use efficiency in rice is only 30%–40%. Excessive N supply leads to nitrate leaching, reduces NUE, and causes agricultural non-point source pollution,including soil acidification and water eutrophication, which explains the ecological degradation in the hilly region of Sichuan under long-term over-fertilization (Sun et al., 2012). Excessive reduction is supply of N (N1) slightly increased NUE compared with N3, but insufficient N supply limited yield and reduced the agronomic benefit of NUE, similar to the conclusion of Liu et al (Liu et al., 2023), who found that optimized nitrogen application (OPT, 240 kg hm^-^²) maintained grain yield (7737 kg hm^-^²) while significantly improving nitrogen-use-efficiency (NUE) and reducing nitrogen leaching. This confirms that NUE improvement must be based on guaranteed yield, and an appropriate N rate of supply is key to synchronizing yield and NUE.This study does not exclude differences in genetic background; instead, it takes advantage of the inherent genetic differences between BU3611 and YX1211 to establish a nitrogen efficiency gradient for comparison. Uniform field cultivation management and fully controlled environmental water and fertilizer conditions were applied to eliminate non-genetic interference, confirming that genetic genotype is the core cause of the differences in nitrogen utilization and grain yield between the two lines.

Significant genotypic differences exist in N accumulation and utilization. Yixiangyou 2115 had slightly higher N accumulation at heading and maturity than Byou 3611, whereas Byou 3611 showed significantly higher NAE, reflecting different genetic advantages in N uptake vs. utilization among N−efficient varieties (Li et al., 2024). The NRE of both varieties under N2 were similarly high (>24%), indicating that appropriate reduction in the rate of supply of N weakened genotypic differences in N recovery and achieved synchronous improvement. This further supports the idea that 150 kg N hm ² is optimal for N conservation and efficiency improvements in this region.

### 4.3 Regulatory effects of the nitrogen application rate on sugar accumulation and sucrose metabolic enzyme activity

Sugar metabolism is the a core process governing photosynthate synthesis, translocation, and accumulation in rice. It coordinates closely with N metabolism to regulate yield and quality (Wang et al., 2022). As a key regulator of sugar metabolism, the N rate significantly affects the soluble sugar and sucrose contents in leaves and grains, and key sucrose metabolic enzymes mediate the regulatory effect of N on sugar metabolism (Zhang et al.,2021). In this study, leaf soluble sugar and sucrose contents increased significantly with increasing application of N, with the highest values under N3, followed by N2 and N0, consistent with the findings of Wang et al (Wang et al., 2022), who confirmed that moderate rates of supply of N increased leaf sucrose phosphate synthase (SPS) activity and enhanced carbohydrate synthesis for grain filling.

As an essential element for photosynthetic enzymes and chlorophyll synthesis (Yang et al., 2020), sufficient N improves leaf chlorophyll content and photosynthetic rate, promoting photosynthate production, whereas N deficiency inhibits photosynthetic system development and reduces leaf sugar synthesis, consistent with the findings of Zhang et al (Zhang et al.,2021). The soluble sugar and sucrose contents first increased and then decreased with increasing N application rates, and N2 was optimal for grain sugar accumulation.Grain sugar decreased significantly under N3, mainly because of a source–sink imbalance and blocked photosynthate translocation to grains (Li et al., 2024). Grain SPS and sucrose synthase (SuSy) activities also decreased under N3, verifying that excessive N supply inhibited the expression of sucrose metabolic enzymes in grains, weakened sink strength, and disrupted source–sink coordination. N2 balanced leaf sugar synthesis and enhanced grain sucrose metabolic enzyme activity, promoting efficient photosynthate translocation and achieving source–sink coordination, similar to the results of Liu et al (Liu et al., 2020). Key sucrose-metabolic enzymes are major targets of N regulation. The N supply rate significantly affected SPS and SuSy activities in leaves and grains, and enzyme changes are highly consistent with sugar contents (Zhang et al., 2021). Leaf SPS activity decreased significantly with decreasing N concentration, in line with leaf sugar variation. However, grain SPS and leaf/grain SuSy activities first increased and then decreased with increasing rate of supply of N and remained high under N2, providing enzymatic support for sucrose translocation and accumulation. This was the main physiological reason for the high grain sugar content under N2. Significant genotypic differences were observed in enzyme activities: Byou 3611 had significantly higher leaf SPS and SuSy activities than Yixiangyou 2115, a core advantage in leaf sucrose synthesis, related to genetic characteristics of sugar metabolism (Li et al., 2024). Variations in the expression of genes encoding sucrose-metabolizing enzymes lead to genotypic differences in enzyme activity. N2 synergistically optimized sucrose-metabolic enzymes in leaves and grains of both varieties, matching leaf synthesis with grain accumulation, which is an important mechanism underlying improved sugar metabolism through appropriate reduction in supply of N. The grain SPS and SuSy activities in 2025 were slightly higher than those in 2024, indicating a minor positive environmental effect without altering the regulatory patterns or genotypic differences, further verifying the stability and suitability of N2.

## 5 Conclusions

This study demonstrated that N application rate is is the dominant factor regulating grain yield, dry matter accumulation, N utilization, and sugar metabolism of rice in the hilly region of Sichuan. Application of 150 kg N hm^-^² (N2) was optimal for Byou 3611 and Yixiangyou 2115. This rate synergistically optimized yield components, including effective panicles and seed-setting rate, enhanced dry matter accumulation and translocation efficiency as well as crop growth rate, and significantly improved N recovery efficiency and agronomic efficiency (NAE of Byou 3611 reached 32.02 kg·kg^-^¹). Stable genotypic differences were observed: Byou 3611 had more effective panicles, a higher seed-setting rate, and stronger dry matter translocation ability,whereas Yixiangyou 2115 had a larger 1000-grain weight and greater post-anthesis dry matter accumulation. Excessive rates of application of N caused luxury N absorption, restricted dry matter translocation, and reduced grain enzyme activity. Too large a decrease in rate of supply of N limited population construction and yield formation.

In summary, for Byou 3611 and Yixiangyou 2115 planted in the hilly region of Sichuan, 150 kg N hm^-^² applied at a ratio of 3:3:4 (basal: tillering: panicle) is recommended. This regime achieves synchronous improvement of high yield, high N use efficiency and ecological safety, providing a theoretical basis and technical support for N−saving and high−efficiency rice cultivation in the region.

## Data Availability

The original contributions presented in the study are included in the article/supplementary material. Further inquiries can be directed to the corresponding authors.
